# Increased cuff-leak pressure after abdominal and spine surgeries: a simple and novel cuff-leak test for tracheal extubation

**DOI:** 10.1007/s00540-025-03487-w

**Published:** 2025-03-25

**Authors:** Takayuki Yamada, Katsuhiko Ishibashi, Yuichi Sakaguchi, Sadatoshi Kawakami, Natsuko Nozaki-Taguchi, Yasunori Sato, Shiroh Isono

**Affiliations:** 1 Department of Anesthesiology, Pain and Palliative Care Medicine, Chiba University Hospital, 1-8-1 Inohana, Chuo-Ku, Chiba, 260-8670 Japan; 2 Department of Anesthesiology, Graduate School of Medicine, Chiba University, Chiba, Japan; 3 Department of Preventive Medicine and Public Health, Keio School of Medicine, Tokyo, Japan

**Keywords:** Anesthesia, Laryngeal edema, Cuff-leak test, Cuff-leak pressure, Extubation

## Abstract

**Purpose:**

Cuff-leak volume (CLV) tests are recommended to avoid extubation failure. We developed a novel cuff-leak pressure (CLP) test that quantitatively assesses upper airway resistance outside the tracheal tube. We hypothesized that CLP (airway pressure during apnea with the cuff deflated under a 6 l/minute oxygen flow) would increase after surgery (primary outcome) and evaluated the accuracy and reproducibility of CLP measurements by measuring the CLV (difference in expiratory tidal volume before and after deflation of a tracheal tube cuff).

**Methods:**

CLV and CLP were measured before and after abdominal surgery (*n* = 25; abdominal group) and cervical spine surgery (*n* = 25; spine group) under general anesthesia and complete neuromuscular blockade.

**Results:**

In both groups, the CLP was significantly higher after surgery (median [25%, 75% interquartile ranges]) (abdominal group: 4.0 [1.0, 8.4] cmH_2_O to 9.0 [3.4, 13.7] cmH_2_O, *P* = 0.007; spine group: 8.0 [3.0, 10.9] cmH_2_O to 11.0 [7.5, 13.5] cmH_2_O, *P* = 0.038). The cutoff values for 100% negative and positive predictive values for a positive CLV test (CLV < 110 ml or 25% of the tidal volume with the cuff inflated) were 12.0 and 17.3 cmH_2_O, respectively, with an AUC of 0.957 (95%CI 0.27–1.20). The CLP and CLV measurements were highly reproducible, as the Kendall’s coefficients of concordance were 0.898 (1st and 3rd) and 0.971 (6 consecutive breaths), respectively, although the CLV progressively increased by 29.0 [1.8, 58.8] ml for the 6 consecutive breaths (*P* < 0.001).

**Conclusion:**

Both the CLP and CLV measurements were accurate and highly reproducible to assess postoperative increase of the upper airway resistance before extubation.

## Introduction

During the process of emergence from general anesthesia and extubation of the tracheal tube, upper airway obstruction is a common respiratory complication possibly resulting in severe life-threatening hypoxemia [1–4]. Although reintubation after general surgery is rare (0.1–0.45%), the incidence rate is 7 to 25 times higher in patients who have undergone head and neck surgery (0.7–11.1%) presumably due to severe postoperative laryngeal edema and upper airway obstruction after extubation [4]. Thus, according to the guideline put forward by the Difficult Airway Society, airway assessments are recommended at the end of surgery and before extubation [5]. However, in a recent review, researchers noted that the guidelines for extubation lack stringent criteria for assessments and decision-making despite the difficulty of predicting the safety of extubation and the significant dependence of its outcome on the intuition of each individual provider [4].

In the operating room, fiberoptic assessments are often performed to predict possible increases in laryngeal resistance after extubation due to recurrent laryngeal nerve palsy and laryngeal edema, particularly in patients undergoing head and neck surgeries [6–9]. However, laryngeal endoscopy requires removal of the tracheal tube, thus impeding secure airway patency; moreover, the fiberoptic bronchoscope is an expensive device and is not available at all institutions. Furthermore, the reproducibility of laryngeal endoscopy among different assessors has not been examined. Other methods, such as laryngeal ultrasonography, have been proposed, but are not widely used in clinical settings possibly due to inadequate assessments of their accuracy and reproducibility [10, 11].

In contrast, in patients under mechanical ventilation in the intensive care unit, the cuff-leak volume (CLV), defined as the difference in expiratory tidal volume before and after deflation of a tracheal tube cuff, is commonly measured prior to tracheal extubation to predict the likelihood of laryngeal stridor and reintubation due to laryngeal edema [12, 13]. However, the CLV does not represent resistance to breathing through the airway bypassed by the tracheal tube and may not reflect severe postoperative laryngeal edema leading to extubation failure in the operating room. In a few previous studies, the researchers measured the CLV in patients after cardiac, spine and Trendelenburg- robotic surgeries [14–17]. While the CLV decreased in all studies, the association between a positive CLV test and extubation failure remained uncertain, possibly due to the insufficient sample size of the studies.

We developed a unique cuff-leak pressure (CLP) test which measures the pressure difference across the upper airway outside the tracheal tube under constant flow. In accordance with flow dynamics theories such as Ohm’s law, the CLP directly reflects the resistance of the upper airway bypassed by the tracheal tube [18]. The CLP values in patients with a small mandible were greater than those in normal patients and decreased after surgical expansion of the small mandible [19]. Although the predictive ability of the CLP test may be equivalent to that of the CLV test because of its association with flow dynamics, we have not measured changes in CLP before and after surgery as a preliminary clinical study for future large-scale outcome studies assessing the predictive ability of CLP for extubation failure in surgical patients.

Therefore, in this exploratory observational study, we 1) hypothesized that the CLP (primary variable) would be higher after surgery (primary outcome), as would the CLV, and 2) evaluated the accuracy and reproducibility of CLP measurements by measuring the CLV in patients undergoing abdominal and cervical spine surgery under general anesthesia with tracheal intubation.

## Methods

This prospective observational and exploratory study was performed in the operating rooms of Chiba University Hospital, Chiba, Japan. Ethical approval for this study (ethical committee number: 3676) was provided by the Ethical Committee of the Graduate School of Medicine, Chiba, Japan (chairperson: Prof. Masaomi Iyo), on March 16, 2020. The study was registered prior to patient enrollment in the University Hospital Information Network (UMIN) Clinical Trial Registry (UMIN000039910; principal investigator: Shiroh Isono; date of registration: March 23, 2020; website: https://upload.umin.ac.jp/cgi-bin/ctr/ctr_view_reg.cgi?recptno=R000045503). Contact information for the full trial information is available on the UMIN website. Written informed consent was obtained from each participant after full disclosure of the aim and potential risks of the study.

Enrollment in this study started on June 8, 2020, and ended on February 4, 2022. Adults (over 20 years old, male and female) who underwent abdominal surgery or cervical spine surgery under general anesthesia were included in the study. We anticipated the development of severe laryngeal edema in patients undergoing cervical spine surgery regardless of the use of either the anterior or posterior approach. Furthermore, we also suspected the development of laryngeal edema, although less severe, in patients who underwent abdominal surgery, particularly when it was performed with the patient in a head-down position. Patients who had a malformed airway, palatine tonsil hypertrophy, severe nasal congestion, tracheostomy, a history of difficult intubation, uncontrolled asthma, pneumonia, intracranial lesions, a history of dialysis treatment, a history of myocardial infarction, and a history of head and neck surgery were excluded.

### Anesthesia management

The anesthesia technique and surgical procedure were not standardized in this study and were determined by the anesthesiologist. General anesthesia was induced with fentanyl, remifentanil and propofol, and complete neuromuscular blockade was achieved via an injection of rocuronium. After confirming complete neuromuscular blockade, a polyvinyl chloride cuffed tracheal tube (a 7.0-mm internal diameter for females and a 7.5-mm internal diameter for males) was inserted into the trachea using either a Macintosh laryngoscope (*n* = 17), videolaryngoscope (McGRATHMAC™, Aircraft Medical, Edinburgh, Britain, *n* = 8; AirwayScope, AWS-S100, HOYA, Tokyo, Japan, *n* = 24), or laryngeal endoscope (MAF-GM2, with a 3.9-mm outer diameter; Olympus, Tokyo, Japan *n* = 1). The depth of neuromuscular blockade was monitored via acceleromyograph neuromuscular monitoring (TOF-Watch; Organon Ireland Ltd., Dublin, Ireland) throughout the procedure.

### Assessments of laryngeal edema immediately before and after surgery

**Fig. 1:**
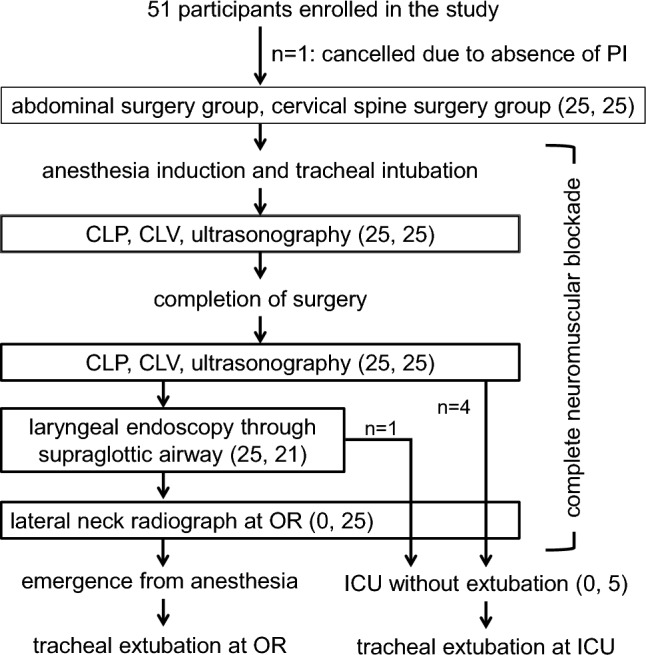
Study protocol for patient enrollment and data acquisition before and after surgery under general anesthesia and complete neuromuscular blockade. *ICU* intensive care unit, *OR * operating room, *PI * primary investigator. The numbers in parentheses represent the number of patients undergoing abdominal or cervical neck surgery and CLP and CLV measurements

Patients were screened for laryngeal edema immediately before the start of surgery and immediately after the end of surgery under anesthesia and complete neuromuscular blockade (a train of four ratio of 0) while in the neutral head and neck position (minimum head elevation with a thin circular cushion while lying spine on a flatbed). After surgery, laryngeal endoscopy was performed using a supraglottic airway device (AuraGain™, Ambu®, Ballerup, Denmark), which was replaced with a tracheal tube in accordance with the technique proposed by Koga et al. [2]. The oropharyngeal cavity was aspirated to minimize the effects of secretions, and the gastric tube was kept in place. Laryngeal endoscopy was not performed via the supraglottic airway if the attending anesthesiologist considered it inappropriate and if cervical spine surgery involving three or more intervertebral disks using the anterior approach was planned. The study protocol is shown in Fig. 1.

### Cuff-leak pressure (CLP) test

**Fig. 2:**
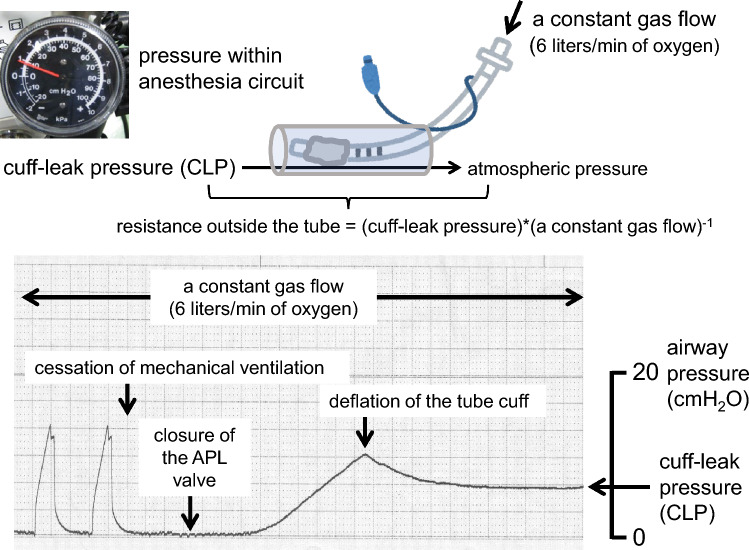
Principle and example of the cuff-leak pressure test. While the cuff of the tracheal tube is deflated, the pressure difference across the upper airway outside the tracheal tube (cuff-leak pressure) is measured under a constant continuous oxygen outflow (6 l/min) delivered from the closed anesthesia breathing circuit. In this situation, the cuff-leak pressure measured at the anesthesia breathing circuit reflects upper airway resistance outside the tracheal tube. As the sample tracing presents, the cuff-leak pressure is determined when the pressure becomes stable

Figure 2 presents the technical details of the CLP test. The CLP represents airway resistance outside the tracheal tube for a constant airflow (resistance = pressure difference/airflow). Owing to the inverse relationship between resistance and the cross-sectional area (resistance ∝ length/area), small but critical airway narrowing can be detected as a larger change in the CLP. First, mechanical ventilation was ceased under a constant pure oxygen flow of 6 l/min through the anesthesia breathing circuit with the tube cuff inflated and the pop-off valve closed. This resulted in a gradual increase in airway pressure measured by a pressure gage (Portex Cuff Inflator Pressure Gauge, Portex Limited, Hythe, Kent, UK) connected at the elbow connector of the breathing circuit, and the tube cuff was deflated when the airway pressure increased to 10 cmH_2_O. Thereafter, the airway pressure either decreased or increased from 10 cmH_2_O and stabilized. Stable airway pressure was defined as the CLP. When the airway pressure increased to over 20 cmH_2_O, the oxygen flow rate was reduced to 3 l/min and the stable airway pressure was measured. The CLP was calculated as twice the value of the airway pressure under 3 l/min. CLP was measured three times before and after surgery.

### Cuff-leak volume (CLV) test

The CLV was measured before and after surgery in accordance with previous studies [6, 20, 21]. In brief, pressure-controlled mechanical ventilation with the tracheal cuff inflated to achieve an expiratory tidal volume of 10 ml/kg-ideal body weight was maintained. The expiratory tidal volume was measured during 6 consecutive breaths using a respiratory monitoring unit of a patient biologic monitoring system (GF-320R, Nihon Kohden Co, Tokyo, Japan) (GF-220R Multigas/Flow unit, Nihon Kohden, Tokyo, Japan). Similarly, the expiratory tidal volume was measured during 6 consecutive breaths immediately after deflation of the tracheal cuff. The CLV was calculated as the difference in the average expiratory tidal volume with the cuff inflated and deflated. The CLV test was defined as positive when it was either less than 110 ml or less than 25% of the tidal volume with the cuff inflated, on the basis of previously-reported cutoff values that successfully predicted adverse outcomes, such as laryngeal stridor and reintubation after tracheal extubation, as evidenced by high specificity and moderate sensitivity [12, 20].

### Laryngeal ultrasonography

With the tracheal tube still inserted into the trachea, laryngeal ultrasonography was performed with an ultrasound machine (CX50; Philips Ultrasound, Tokyo, Japan), using a linear probe (6–12 MHz) in accordance with a previous report [10, 11]. The probe was placed on the cricothyroid membrane under guidance of a transverse view of the larynx. All patients underwent standard scanning, which involved the ultrasound probe being placed in the transverse plane on the cricothyroid membrane for visualization of the true vocal cords, false cords, thyroid cartilage, and arytenoid cartilage. The laryngeal air-column width was defined as the width of air passing through the vocal cords. The air-column width was recorded during cuff inflation and deflation over three consecutive respiratory cycles. The difference in the laryngeal air-column width between tracheal cuff inflation and deflation was measured and averaged for analysis in this study. A laryngeal air-column width less than 0.8 mm was reported to be the cutoff for an increased risk of upper airway obstruction after extubation in a previous study [9].

### Laryngeal endoscopy

Laryngeal endoscopy was performed using a supraglottic airway device after removal of the tracheal tube [2]. A thin endoscope (LF-DP, 3.1 mm outer diameter; Olympus, Tokyo, Japan, or F1-9RBS; Pentax, Tokyo, Japan.) was inserted through an elbow connector to the supraglottic airway device to visualize the vocal cords. Endoscopic images of the vocal cords and surrounding laryngeal structure were recorded for later analysis of laryngeal edema and the vocal cord angle. Three certificated anesthesiologists, who were blinded to the patient’s background information and study purpose, reviewed images of the laryngeal structures and scored the laryngeal edema 0 to 4, with 0 indicating no injury or hyperemia; 1 (mild) indicating supraglottic edema; 2 (moderate) indicating both supraglottic and glottic edema and/or vocal process granuloma, vocal cord ulcers and/or arytenoid luxation; 3 (severe) indicating more intense supraglottic and glottic edema with or without hematomas; and 4 (very severe) indicating subglottic lesions and vocal cord dysfunction [9].

### Primary outcome, sample size calculation and statistical analysis

We tested our hypothesis that the new-developed CLP (primary variable) would be higher after surgery (primary outcome). In our preliminary study, CLP was measured in patients who underwent abdominal surgery (*n* = 19: an increase of 0.9 ± 2.4 cmH_2_O from 5.6 ± 3.2 cmH_2_O before surgery) and those who underwent cervical spine surgery (*n* = 28: an increase of 8.1 ± 7.8 cmH_2_O from 7.7 ± 4.2 cmH_2_O before surgery). As we planned to enroll more patients who underwent abdominal surgery in the head-down position in this study, we expected that the CLP after surgery would be twofold higher than that before surgery (2 cmH_2_O). The sample size was calculated to ensure that an increase in the CLP of more than 2 cmH_2_O in abdominal surgery patients would be detected, resulting in a larger sample size. Assuming that α = 0.05 (two tailed) and β = 0.8, the suitable sample size was 23 patients (SigmaPlot 14.5; Systat Software, Point Richmond, CA). The appropriate sample size was set to 25 patients for both the abdominal surgery group and the cervical spine surgery group.

Continuous variables are presented as medians [25%, 75% interquartile ranges], and categorical variables are presented as frequencies and percentages. No intervention for managing missing values was planned. Primary and secondary outcomes before and after surgery were compared with the Wilcoxon signed rank test. Correlations between the CLP and CLV were analyzed via Pearson’s correlation coefficient. Reproducibility was assessed via Kendall’s coefficient of concordance for repeated measurements of the CLP, CLV and difference in the laryngeal air-column width and by the kappa statistic for the laryngeal endoscopy scale among different assessors. Friedman repeated measures analysis of variance on ranks was used to evaluate possible significant changes in consecutive CLP and CLV measurements and to assess possible differences in laryngeal endoscopy scales scores among assessors. The cutoff values for CLP to predict positive and negative CLV test results were determined using the Youden index from the receiver operating characteristic (ROC) curve. To select patient and surgical procedure factors that independently contribute to postoperative CLP values, we used the Schwarz-Bayes criterion (SBC) to select the final multivariable linear regression model. All the candidate explanatory variables were evaluated, and the model with the smallest SBC value was chosen as the final model. This approach ensures that the selected model achieves an optimal balance between goodness-of-fit and complexity, thereby minimizing the risk of overfitting. The final model was refit with 100,000 bootstrap samples to provide the final coefficients which were determined by calculating the mean values of the coefficients across all bootstrap samples. This approach was chosen because it provides a straightforward estimation that captures the central tendency of the bootstrap distribution. A P value < 0.05 indicated statistical significance, and all P values were two -sided. All the statistical analyses were performed by Y.S. and S.I. using SAS software version 9.4 (SAS Institute, Cary, North Carolina, USA) and SigmaPlot 14.5 (Systat Software Inc., Point Richmond, CA).

## Results

Fifty-one patients who met the inclusion and exclusion criteria were invited, and written informed consent was obtained from each participant (Fig. 1). Clinical data were collected from 50 participants, and one patient was excluded on the day of surgery by the primary investigator (T.Y.). The characteristics and perioperative management of each group of participants are presented in Table 1. The tracheal tube was successfully removed in the operating room in all participants, except for 5 patients who underwent anterior cervical spine fixation surgery as planned. For these 5 patients, tracheal extubation was planned and successfully achieved in the intensive care unit. All measurements were performed without incident. No desaturation occurred as a result of apnea during the process of mechanical ventilation discontinuation for CLP tests.

**Table 1:** Patient characteristics and perioperative management

	Cervical spine surgery group (*n* = 25)	Abdominal surgery group (*n* = 25)
*Patient characteristics*		
Age (year)	71 [58, 78]	72 [68, 77]
Sex (male, female)	(10, 15)	(8, 17)
Weight (kg)	57.0 [49.8, 66.0)]	66.0 [53.6, 70.6]
Height (m)	1.58 [1.51, 1.64]	1.66 [1.58, 1.70]
Body mass index (kg/m^2^)	22.9 [20.9, 26.0]	23.4 [21.8, 25.2]
ASA-physical status (1, 2, 3)	(1, 19, 5)	(3, 18, 4)
Hypertension	14 (56)	12 (84)
Diabetes mellitus	8 (32)	9 (36)
Ischemic heart disease	2 (8)	0 (0)
COPD	0 (0)	2 (8)
Chronic kidney disease	3 (12)	1 (4)
Obstructive sleep apnea syndrome	11 (44)	7 (28)
Mallampati class (1, 2, 3)	(16, 4, 3)	(8, 8, 1)
STOP-BANG score (0, 1, 2, 3, 4, 5)	(1, 1, 9, 9, 4, 1)	(3, 4, 3, 7, 4, 3)
*Perioperative managements*		
Tracheal tube diameter (7.0 mm, 7.5 mm)	(11, 14)	(8, 17)
Duration of anesthesia (min)	328 [275, 584]	361 [316, 481]
Duration of the surgery (min)	222 [147, 435]	282 [244, 381]
Fluid infusion volume (ml)	1550 [1315, 2916]	2100 [1685, 2460]
blood loss (g)	50 [0, 150]	90 [13, 190]
Urine output (ml)	425 [220, 985]	235 [163, 355]
Laparoscopic surgery	N/A	19 (76)
Head-down posture	N/A	10 (40)
Prone position	17 (68)	N/A

Values are presented as medians [25%, 75% interquartile ranges] or numbers (%)

### Changes in CLP and CLV after surgery

The median CLP significantly increased from 4.0 [1.0, 8.4] cmH_2_O before surgery to 9.0 [3.4, 13.7] cmH_2_O after surgery in the abdominal surgery group (*P* = 0.007), and from 8.0 [3.0, 10.9] cmH_2_O before surgery to 11.0 [7.5, 13.5] cmH_2_O after surgery in the cervical spine surgery group (*P* = 0.011), supporting the primary hypothesis (Table 2). The CLV was significantly lower after abdominal surgery, but not after cervical surgery. Pearson’s correlation analysis indicated strong correlations between the CLP and CLV (*n* = 100, *r* = 0.747).

**Table 2:** Results of assessments for laryngeal edema before and after surgery with various techniques

	Cervical spine surgery group (*n* = 25)		*P* value	upper abdominal surgery group (*n* = 25)		*P* value
	Before	After		Before	After	
Cuff-leak tests	*n* = 25	*n* = 25		*n* = 25	*n* = 25	
Cuff-leak volume (ml)	413 [192, 521]	261 [191, 426]	0.11	524 [416, 555]	319 [196, 496]	0.002
% Cuff-leak volume (%-tidal volume)	77.1 [37.0, 96.8]	54.1 [34.0, 78.6]	0.054	85.2 [62.3, 90.7]	55.0 [23.1, 82.9]	0.001
Cuff-leak pressure (cmH_2_O)	8.0 [3.0, 10.9]	11.0 [7.5, 13.5]	0.011	4.0 [1.0, 8.4]	9.0 [3.4, 13.7]	0.007
Laryngeal ultrasonography	*n* = 23	*n* = 16		*n* = 25	*n* = 25	
Difference of air-column width (mm)	1.1 [1.1, 1.2]	1.1 [1.0, 1.1]	0.720	1.2 [1.1, 1.2]	1.1 [1.0, 1.2]	0.016
Laryngeal endoscopy Scale score (0, 1, 2, 3, 4)		*n* = 21			*n* = 25	
Assessor #1	N/A	1.0 [1.0, 2.0]		N/A	1.0 [0, 1.0]	
Assessor #2	N/A	3.0 [2.0, 3.0]		N/A	2.0 [1.0, 3.0]	
Assessor #3	N/A	1.0 [0, 1.5]	N/A	N/A	0 [0, 2.0]	N/A

Values are presented as medians [25%, 75% interquartile ranges]

### Performance of the CLP test for predicting positive CLV test

**Fig. 3:**
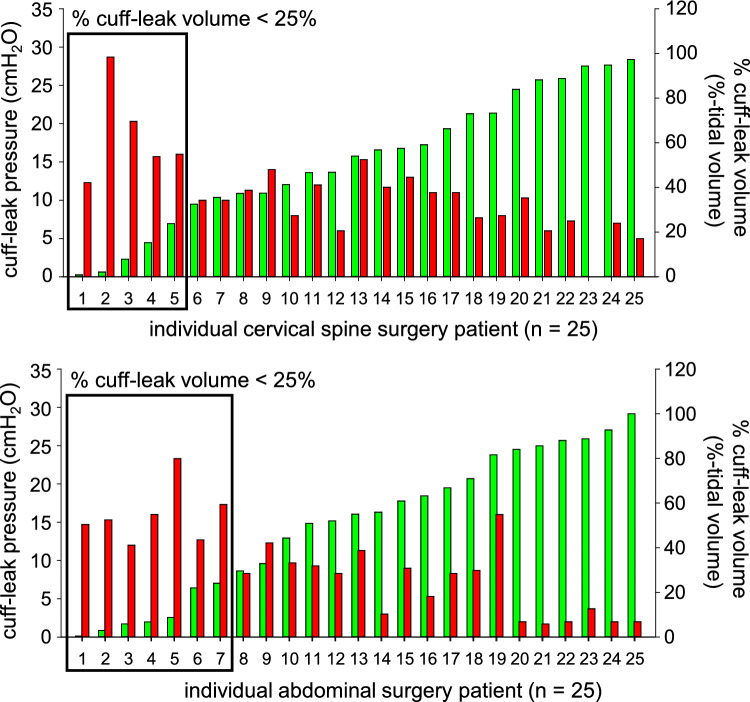
Individual postoperative cuff-leak pressure (red bar) and cuff-leak volume (green bar) for patients who underwent cervical spine surgery (*n* = 25, upper panel) and patients who underwent abdominal surgery (*n* = 25, lower panel) are presented. Patients who had a positive cuff-leak volume test result were defined as having a cuff-leak volume less than 110 ml or 25% of the tidal volume are enclosed in squares. Notably, significantly greater cuff-leak pressures were detected in patients who had a positive cuff-leak volume test

**Fig. 4:**
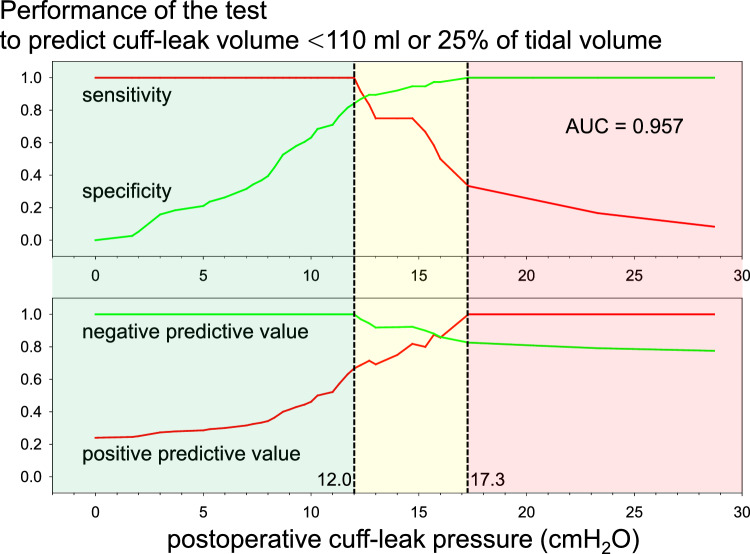
The results of a receiver operating characteristic analysis for predicting a positive cuff-leak volume test, defined as a cuff-leak volume less than 110 ml or 25% of the tidal volume, are summarized as a function of different postoperative CLP values. Note the 100% negative predictive value below 12.0 cmH_2_O (green zone) and the 100% positive predictive value above 17.3 cmH_2_O (red zone). These cutoff values could be used as clinical indicators for safe tracheal extubation and cautious tracheal extubation or delayed tracheal extubation, respectively

The postoperative CLP and CLV values for each group are presented in Fig. 3. Among the 50 participants, 12 had a postoperative CLV that was either less than 110 ml or less than 25%. The median postoperative CLP values in the patients with a positive CLV test (*n* = 12) were significantly greater than those in the patients with a negative CLV test (*n* = 38) (15.9 [13.2, 19.6] cmH_2_O versus 8.3 [5.2, 11.1] cmH_2_O, *P* < 0.001). The postoperative CLP values were indirectly associated with the postoperative CLV values (*n* = 50, *r* =  – 0.716, *P* < 0.001). The area under the curve of the ROC curve for detecting a positive CLV test with the CLP value was 0.957 (95%CI 0.27–1.20). Differences in the sensitivity, specificity, positive predictive value, and negative predictive value for different postoperative CLP values are plotted in Fig. 4. The threshold for a 100% negative predictive value was 12 cmH_2_O CLP resulting in 100% sensitivity. The threshold for a 100% positive predictive value was 17.3 cmH_2_O resulting in 100% specificity.

### Performance of laryngeal ultrasonography for assessing postoperative laryngeal edema and predicting positive CLV test

The difference in the air-column width between tracheal cuff inflation and deflation was successfully measured via laryngeal ultrasonography in 25 abdominal and 16 cervical spine surgery patients, excluding 9 patients who underwent anterior cervical fixation surgery. It significantly decreased by 0.1 mm after surgery in the abdominal surgery group, but not in the cervical spine surgery group (Table 2). The difference in the air-column width was not below 0.8 mm in either group. The median values of the difference in the postoperative air-column width in patients with a positive CLV test (*n* = 10) were not different from those in patients with a negative CLV test (*n* = 38) (1.0 [1.0, 1.3] mm versus 1.1 [1.0, 1.1] mm, *P* = 0.438).

### Performance of laryngeal endoscopy for predicting positive CLV test

**Fig. 5:**
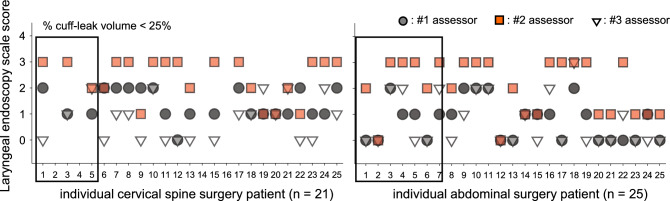
Individual laryngeal endoscopy scale scores determined by three different assessors for patients undergoing cervical spine surgery (*n* = 21, upper panel) and patients undergoing abdominal surgery (*n* = 25, lower panel) are presented. Patients who had a positive cuff-leak volume test were defined as those whose cuff-leak volume was less than 110 ml or 25% of the tidal volume and are enclosed in squares. Note the significant variability among the assessors

Laryngeal endoscopy after surgery was performed in 46 participants, excluding 4 patients who were not extubated in the operating room after undergoing anterior cervical spine fixation surgery. The laryngeal endoscopy scale scores determined by three anesthesiologists (assessors #1, #2, and #3) are presented in Table 2 and Fig. 5. The postoperative laryngeal endoscopy scale scores of patients with a positive CLV test (*n* = 10) were not significantly different from those of patients with a negative CLV test (*n* = 38) (0.5 [0, 2.0] versus 0 [0, 1.0], *P* = 0.630).

### Reproducibility of the various techniques for assessing laryngeal edema

CLP measurements were highly reproducible, as evidenced by their Kendall coefficients of concordance of 0.916 (1st and 2nd), 0.898 (1st and 3rd), and 0.950 (2nd and 3rd). No significant change in repeated CLP measurements was detected, with only small differences among the measurements (< 0.1 cmH_2_O) (*P* = 0.754). Kendall’s coefficients of concordance of all CLV values obtained at each of the 6 consecutive breaths were above 0.971. However, the CLV progressively increased by 29.0 [1.8, 58.8] ml for the 6 consecutive breaths (*P* < 0.001). Kendall’s coefficients of concordance of laryngeal ultrasonography were 0.176 (1st and 2nd), 0.301 (1st and 3rd), and 0.261 (2nd and 3rd), indicating low reproducibility of the clinical test results. As clearly presented in Fig. 5, the laryngeal endoscopy scale scores differed among assessors, even for patients with a positive CLV test. Compared with assessors #1 (1.0 [0, 2.0]) and #3 (0 [0, 2.0]) (*n* = 46, *P* < 0.001), assessor #2 (3.0 [1.0, 3.0]) reported significantly higher laryngeal endoscopy scale scores. The reproducibility of the laryngeal endoscopy scale score among assessors #1, #2 and #3 was quite low, as the kappa statistic values between #1 and #2, #1 and #3, and #2 and #3 were -0.001, 0.16, and 0.02, respectively.

### Independent risk factors for an increased postoperative CLP

A multivariable linear regression model was created to explain logarithmic transformation of postoperative CLP values using the patient and surgical variables listed in Table 3. Three variables (higher body mass index, prone or head-down position, and larger urine output) were identified as statistically significant independent predictors for greater postoperative CLP (*R*^2^ = 0.292, *P* = 0.001).

**Table 3:** Results of the multivariable linear regression analysis of postoperative cuff-leak pressure

Dependent variable	Independent risk factors	Estimate	Standard error	*P* value
Log (postoperative cuff-leak pressure) (cmH_2_O), R^2^ = 0.292	Intercept	0.298	0.587	0.614
	Body mass index (kg/m^2^)	0.061	0.023	0.012
	Prone or head-down position	0.395	0.176	0.03
	Urine output	0.0005	0.0002	0.008

## Discussion

In this prospective observational study, we found that (1) CLP was significantly higher after surgery, (2) both the CLV and CLP measurements were highly reproducible, although the CLV progressively increased during consecutive breaths, (3) a postoperative CLP less than 12.0 cmH_2_O and greater than 17.3 cmH_2_O revealed 100% negative and positive predictive values for predicting a CLV less than 110 ml or 25% of the tidal volume, (4) other clinical tests assessing postoperative laryngeal edema, such as laryngeal endoscopy and laryngeal ultrasound, were not reproducible and did not reveal any differences in the results after surgery, and (5) a higher body mass index, prone or head-down position, and greater urine output were independent risk factors for an increased postoperative CLP.

### Clinical value of CLP measurements

We consider that there are several methodological and clinical advantages of the CLP test over other clinical tests. First, the CLP reflects the resistance of the upper airway outside the tracheal tube under constant oxygen outflow (Fig. 2). The CLV not only reflects upper airway resistance outside the tracheal tube but is also influenced by the tidal volume, which is affected by the patient’s body size, ventilation mode, and pulmonary mechanics. Despite these limitations, the clinical performance of the CLV test for predicting extubation failure in intensive care unit patients has been established. The discrepancy between its physiologic meaning and clinical usefulness motivated us to test the performance of the CLP test, as its results more accurately reflects upper airway patency. In critical laryngeal edema, small differences or changes in the airway cross-sectional area could result in diverse outcomes after extubation. Small differences in the cross-sectional area could be detected by large differences in airway pressure across the airway because of their inverse relationship. This feature of the CLP is in significant contrast to that of the CLV, in which a small difference in the cross-sectional area is unlikely to be determined by a small difference in the CLV. Furthermore, a significant increase in CLV measurements during consecutive breaths was found in this study. These methodological disadvantages of the CLV test, particularly in patients with suspected laryngeal edema, may explain the relatively wide range of CLV cutoff values for the prediction of extubation failure, as indicated by a recent systematic analysis (50–283 mL: median, 110 mL) [13]. In contrast, lower and higher CLP values may be used as indicators for safe and high-risk tracheal extubation, as we succeeded in determining the CLP cutoff values of 12.0 and 17.3 cmH_2_O for negative and positive CLV tests, respectively (Fig. 4).

Second, CLP measurements require only an oxygen flow meter and pressure gage equipped in a standard anesthetic machine and can be performed in any operation room or setting where these devices are available. Although previous studies revealed the possible usefulness of laryngeal endoscopy and ultrasonography for assessing laryngeal edema [9, 22], our results did not support their reproducibility and did not reflect postoperative changes in laryngeal airway patency. We consider that these tests alone are insufficient for diagnosing laryngeal edema and must be combined with other tests for a comprehensive diagnosis [4]. Despite the advantages of CLP measurements, we only tested their predictive ability by comparing them with CLV measurements in this study, and researchers need to assess their clinical usefulness for predicting successful tracheal extubation and the occurrence of respiratory complications such as stridor and reintubation in future studies.

### Mechanisms of an increased CLP after surgery

While pharyngeal airway obstruction due to inadequate recovery of consciousness after extubation could be resolved using airway maneuvers, insertion of a nasal airway and application of continuous positive airway pressure, mechanical laryngeal airway obstruction after extubation could develop even in fully awake patients requiring more invasive treatments, such as reintubation [23]. As a major methodological limitation, cuff-leak tests measuring either volume or pressure are unable to identify the site of limited airflow and increased resistance along the upper airway. We assume that laryngeal edema is the dominant mechanism for an increased postoperative CLP in most postoperative patients immediately after surgery since the cross-sectional area at the vocal cords is the smallest along the upper airway. However, postoperative upper airway obstruction can occur anywhere along the airway. In fact, patient and surgical factors such as obesity, obstructive sleep apnea, upper airway abnormalities, invasive head and neck surgery, massive fluid infusion, prolonged surgery, and a head-down position may exacerbate tissue edema anywhere along the upper airway leading to pharyngeal as well as laryngeal airway narrowing [1, 4]. In this study, we also identified independent patient and operative risk factors explaining the higher postoperative CLP, which is in agreement with previous studies revealing the risk factors for a positive CLV test [24–26]. Furthermore, cuff-leak tests are performed under anesthesia and complete neuromuscular blockade, both of which impairs pharyngeal airway patency and improves the laryngeal airway patency [27]. Accordingly, reassessments of the CLP and volume after recovery of neuromuscular function and consciousness when improvement in pharyngeal airway patency is expected to be helpful for determining the optimal timing of tracheal extubation. Alternatively, evaluation of the upper airway via flexible endoscopy and laryngoscopy may provide supplementary information for determining the optimal timing of tracheal extubation.

### Limitations of the study

In addition to the methodological limitations of the CLP test discussed above, the study design warrants careful interpretations of the results of the study. First, as this study was observational in nature, we did not test the clinical usefulness of the tests for predicting the occurrence of stridor and reintubation after extubation. Furthermore, the CLV test is limited to patients in the intensive care unit under long-term mechanical ventilation, as the clinical usefulness of the test has not been systematically tested and is therefore unvalidated in patients at high risk for complications associated with extubation in the operating room. This important clinical question needs to be addressed by randomized controlled trials comparing the incidence of extubation failure with or without cuff-leak tests in a large sample of patients at risk for postoperative upper airway obstruction. Second, changes in the laryngeal ultrasonography and endoscopy scale scores insignificantly differed from those reported in previous studies [6–11]. The disagreements may be multifactorial, but partly explained by difficulty in performing accurate and reproducible measurements and assessments. In fact, laryngeal ultrasonography is not routinely performed at our institution, as laryngeal endoscopy is preferred for patients at risk for severe laryngeal edema in our institution. Laryngeal endoscopy via the supraglottic airway improves the safety of assessments; however, laryngeal endoscopy via the supraglottic airway may be hindered by limited visibility of the observation field leading to different laryngeal endoscopy scale scores [28]. Third, owing to the exploratory nature of this study, we did not correct for multiplicity in the multivariable analysis performed to identify variables likely related to the increased postoperative CLP. This may increase the chance of a type I error, while the selected variables are clinically relevant and may serve to produce hypotheses to be tested in future clinical studies. Finally, the mechanisms underlying the increased CLP after abdominal and spine surgery observed in this study were not clarified in this study. Future studies assessing the location(s) and pathophysiological mechanisms associated with an increased CLP would improve the safety of airway management under anesthesia.

In conclusion, both CLP and CLV measurements are accurate and highly reproducible in patients undergoing abdominal and cervical spine surgery. The CLP test may be an alternative and reliable reproducible clinical test that can be performed immediately before extubation in the operating room in patients at risk for postoperative upper airway obstruction.
